# Personality Traits of Profoundly Hearing Impaired Adolescents with Cochlear Implants – A Comparison with Normal Hearing Peers

**DOI:** 10.3389/fpsyg.2018.00161

**Published:** 2018-02-20

**Authors:** Merle Boerrigter, Anneke Vermeulen, Henri Marres, Margreet Langereis

**Affiliations:** ^1^Department of Otorhinolaryngology, Radboud University Medical Center, Nijmegen, Netherlands; ^2^Donders Institute for Brain, Cognition and Behaviour, Radboud University, Nijmegen, Netherlands

**Keywords:** cochlear implant, hearing loss, personality, adolescence, speech perception, language comprehension

## Abstract

The aim of this study was to compare the personality traits of adolescents with cochlear implants (CIs) to a reference group (normal-hearing peers). In the past, the personality development of hearing impaired adolescents was severely compromised. Improved speech perception with CI significantly increased their perspectives. In addition, differences between the reference group and CI users were investigated on personality traits according to level of speech perception skills (high/low) and level of language comprehension (adequate/poor). A cohort of 59 adolescents was assessed 10 years after CI implantation. Personality traits were measured using the standardized Dutch Personality Questionnaire, which consists of 5 scales: Inadequacy, Social Inadequacy, Recalcitrance (RE), Perseverance, and Dominance. Speech perception and language comprehension were tested with standardized tests. The distributions of personality scores, in the clinical or non-clinical range, for the CI group were compared to the reference group using the Chi-Square test for Goodness of Fit. Adolescents with CI showed normal or favorable distributions on all personality scales except for the RE scale. There was a significant influence of speech perception and language comprehension on this scale. Consequently, adolescents with CI who demonstrated high speech perception and adequate language comprehension scores showed similar distribution patterns as the reference group on all personality scales. In conclusion; personality traits that reflect social relations, self-conscience, and school- and task orientation in adolescents with CI are similar to those in normal-hearing peers. This holds, despite variations in speech perception ability and language comprehension levels, for the CI group. On the RE scale, the adolescents with CI with low speech perception and poor language comprehension scores are more likely to score in the clinical deviant range and are at risk.

## Introduction

Profound hearing impairment (HI), from birth or early childhood has a lifelong influence on communication, language development, mental health, and social and emotional wellbeing (Cambra, 1996; de Graaf and Bijl, 2002). Limitations in hearing and (secondary) problems in communication and language development negatively affect the mental health of profoundly HI children with hearing aids (Polat, 2003). The majority (95%) of HI children are born in hearing families with aural communication as the main form of communication (Mitchell and Karchmer, 2004). As a consequence, the social, home, and community environments are mainly oriented toward auditory-based communication. Even with the most powerful hearing aids, children with a profound HI have no auditory access to environmental sounds, speech sounds, and spoken language. Environmental information that is limited or is misinterpreted, results in a world that may seem unpredictable and threatening to a young person. Thus, hearing loss effects the information about social relations, such as cause and consequence. The impact of a combined sensory and communicative impairment, such as profound hearing loss, on social-emotional and psychosocial development therefore, is considerable.

Social and emotional development includes the development of personality traits. A social acceptable development of personality traits and behavior corresponds to expectations of the social environment. These expectations are called developmental tasks and include developing autonomy, achieving emotional independence, developing close relationships with peers, achieving socially responsible behavior, and achieving emotional stability (Sawyer et al., 2012; Pinquart and Pfeiffer, 2014). During the transition from childhood to adolescence, one’s personality develops and personality traits are defined (Denham et al., 2009). Personality stabilizes in adulthood (Roberts and DelVecchio, 2000). The transition to adolescence is, due to achieving all the developmental tasks, a challenging period for hearing adolescents. It is expected that development of personality traits in adolescents with a profound HI will pose additional challenges and places this group at risk for developing disordered personality traits.

However, the auditory and communication prospects for most profound HI children have improved since the 1990s. Due to the application of cochlear implants (CIs), which provide auditory input via electrical stimulation of the cochlea, even profoundly HI people can access environmental sounds, hear their own speech and the spoken language of others. Thus, it is to be expected that the application of CI would prevent the development of disordered personality traits.

Research on the effect of hearing loss on the development of personality traits is scarce. The attainment of developmental tasks, such as social responsible behavior and close relationships with peers, can be complicated by hearing loss (Kluwin et al., 2002). Studies of social emotional development and mental health have been carried out. Hearing status seems to play a role in developing socially responsible behavior. For instance, rather than modeling problem solving, parents are more likely to model avoidance and physical action as methods for solving problems or they tend to solve social problems for their HI child because of the child’s difficulty communicating. As a result, a HI child is likely to have fewer opportunities to learn from the social situation. The child is unaware how his/her behavior affects others and what alternative behavior could be considered (Vaccari and Marschark, 1997; Calderon and Greenberg, 2003). Therefore, the expectations are that HI might play a role in developing a postponement and avoidance personality trait, where an individual shows little or no responsibility for his/her actions. The HI can also negatively influence the development of emotional stability and achievement of emotional independence from parents. Research by van Gent et al. (2012) and Wiefferink et al. (2012) showed that children with HI had difficulties using strategies to regulate their emotions and using appropriate social skills compared to hearing peers. Other research shows that adolescents and young adults with HI were less confident and more anxious and dejected than normal-hearing peers and experienced feelings of insufficiency and vulnerability (Filipo et al., 1999; van Eldik et al., 2004). This might play a role in developing an insecure, dejected, and despondent personality trait. Communication problems in adolescents with HI are associated with lower levels of self-perceived social acceptance and less close friendships (van Gent et al., 2011, 2012). Moreover, the personality traits of adults with HI were considered more dependent, less confident, less communicative, passive, egocentric, and more aggressive than people without a sensory disability (Cambra, 1996; Nasralla et al., 2009; du Feu and Chovaz, 2014).

The positive influence of CI on speech perception and production, language development, and reading comprehension is well established (Svirsky et al., 2004; Vermeulen et al., 2007; De Raeve, 2010; Kral and O’Donoghue, 2010; Niparko et al., 2010). However, on complex linguistic and verbal cognitive tasks children with CI lagged behind their hearing peers (Kral and O’Donoghue, 2010; Chilosi et al., 2013; Boons et al., 2013a,b; De Raeve et al., 2015). This means that these children have less access to linguistic social and emotional information compared to normal hearing peers. So CI users have limited possibilities to achieve and understand the auditory refinements of social and emotional language (Calderon and Greenberg, 2003). A study of Wiefferink et al. (2012) showed that children with CI lag behind on some aspects of emotion regulation and social functioning compared to their normal-hearing peers. The children with CI tended to be less socially competent, less able to divert their attention and express negative emotions more often and more intensely. Language skills seem to be positively correlated with emotion regulation and social functioning. CI children with stronger language skills tend to have stronger social competence skills and fewer negative external behaviors than CI children with less-developed language skills.

In the normal hearing population, the auditory system is adapted to integrate information from both ears. This is referred to as binaural hearing, which enables sound localization and improves the ability to detect sounds at lower levels in noisy environment (Akeroyd, 2006). For children with unilateral CI (UCI), speech perception is hindered by background noise, such as in classrooms (Sparreboom et al., 2012; Sarant et al., 2014). Therefore, they benefit less from incidental learning situations like overhearing a conversation between peers. Children with bilateral CI (BICI) perform significantly better than children with UCI on tests of sound localization and speech perception in noise, however not as well as normal-hearing peers (Lovett et al., 2010). These better auditory skills of children with BICI result in better receptive vocabulary and significant higher verbal intelligence than in UCI peers, even comparable to levels obtained by hearing peers (Sarant et al., 2014; Sparreboom et al., 2014; De Raeve et al., 2015; Jacobs et al., 2016). Children with BICI show less behavioral problems than severely HI children with hearing aids and have comparable levels of empathy and social competence as normal hearing peers (Ketelaar et al., 2013; Theunissen et al., 2014). However, peer problems were still experienced by adolescents with CI (Huber et al., 2015).

Based on the considerable improvement in auditory prerequisites for development of social skills, a positive effect on personality development is expected. Therefore, in this study, we investigated the personality traits of profoundly HI adolescents with CI. The hypothesis was that the personality traits of adolescents with CI with relatively high speech perception scores and/or adequate language comprehension scores would be comparable to those of the reference group (normal-hearing peers).

## Materials and Methods

### Participants

The data was collected during the clinical evaluation procedure that routinely occurs at 10 years post-implantation. All subjects who were able to perform the standardized protocol were examined according to clinical presentation order. Data included 59 eligible participants. Descriptive statistics of the participants are listed in **Table 1**. The study group was a heterogeneous group; age at onset of profound hearing loss and age at implantation ranged substantially. The vast majority of the children (88%) had no functional residual hearing prior to cochlear implantation. A small number of children (12%) had a progressive hearing loss and benefited from the use of hearing aids pre-implant. These children received their implants at a relatively high age. The high age at implantation and long duration of hearing loss were unfavorable compared to the current demographics of implanted children, but were current at the time these subjects received implants. The results of the participants were compared to the results of the standardized reference group of The Junior Dutch Personality Questionnaire (Junior Nederlandse PersoonlijkheidsVragenlijst, NPV-J). This reference group contains 3194 participants with a mean age of 13.4 years (SD = 1.6). A total of 48% was male and 52% female. Scores were controlled for gender differences. The test protocol and use of data for scientific purposes was explained to all participants and described in the written evaluation reports for the patients. Informed consent was obtained in all participants. No specific ethical approval was required for this study, in accordance with regulations in the local University Medical Center and Dutch Ethical Standards.

**Table 1:** Descriptive statistics of the participants (*n* = 59).

		*n*	%
Gender	Female	34	57.6
	Male	25	42.4
Unilateral or bilateral CI	Unilateral	50	84.7
	Bilateral	9	15.3
Educational setting	Mainstream	32	54.2
	Specialized for HI	27	45.8
Additional developmental and behavior problems	No	46	78.0


	Yes	13	22.0
	
		**Mean (*SD*)**	**Range**
	
Age at testing		14.32 (2.39)	10.71–20.88
Age at implantation		3.65 (2.06)	0.68–10.08
Duration of deafness before implantation		3.13 (2.14)	0.29–9.77


### Assessments

An audiologist, speech language pathologist, and a psychologist collected measures on auditory speech perception, language comprehension, and personality, respectively, using the tools described in the Section “Auditory Speech Perception.” The order of the three assessments was randomized for each subject.

#### Auditory Speech Perception

Auditory speech perception abilities were assed using a standard Dutch open set identification test, containing consonant – vowel – consonant words (Bosman and Smoorenburg, 1995). This test was carried out in a sound-treated booth. Stimuli were presented in the sound field at a presentation intensity of 65 dB SPL. Scores are expressed as a percentage of correctly recognized phonemes. A score ≥ 85% reflects a high level of speech perception for HI children with CI and is comparable to those of children with a moderate hearing loss, bilaterally fitted with hearing aids, who obtain an average language level (Hicks and Tharpe, 2002).

#### Language Comprehension

Language comprehension *z*-scores were derived from two different assessments: the Reading Comprehension Test (Aarnoutse, 1990) and the Peabody Picture Vocabulary Test-III-NL (PPVT) (Dunn and Dunn, 2005). The PPVT became available in Dutch in 2013. Receptive vocabulary (word comprehension) is known to be an important factor in, and is strongly associated with, reading comprehension for hearing children (Aarnoutse and van Leeuwe, 1988; De Jong and Van der Leij, 2002) as well as for HI children (Marschark and Harris, 1996). The outcomes are expressed in *z-*scores. A *z*-score ≥-1.00 indicates a performance within or above the average range of the reference group and is considered to represent an age adequate score.

#### Personality Traits

The Junior Dutch Personality Questionnaire NPV-J (Luteijn et al., 2005) was used to measure personality traits of the participants. The questionnaire is a standardized diagnostic tool for the detection of clinically deviant personality traits. It is divided into five scales: Inadequacy (IN), Perseverance (PE), Social Inadequacy (SI), Recalcitrance (RE), and Dominance (DO). The intercorrelations between scales support the validity of the instrument. Scales represent relatively independent domains. Each scale contains a series of statements such as, ‘I like being alone.’ The answer options are ‘yes,’ ‘no,’ or ‘I don’t know.’ Every answer is attributed 0, 1, or 2 points. Scale scores were obtained by summing the scores of all questions belonging to a scale. Scale scores were compared with the reference group of the NPV-J. A lower score is favorable for all scales except PE and DO. For PE, a higher score represents a more favorable outcome and for DO, extremes (high or low) are less favorable outcomes. The psychologist supported all participants (in sign or spoken language) to ensure participants understood the questions.

The personality questionnaire is used to identify personality traits in or outside the expected range. Outcomes were classified in average scores or positive or clinical deviant scores (an average range is between μ-1σ or μ+1σ). Clinical deviant scores indicate dysfunctional personality traits. A high IN scale score is associated with an insecure, over-sensitive, dejected, and despondent personality trait and is stated as a clinical deviant score. A high score on the PE scale is associated with high responsibility for schoolwork and a reliable and orderly personality trait and is stated as a positive deviant score.

Adolescents with a positive deviant score on this scale are described as competitive. They can concentrate relatively long and good and work neatly. A low score on the PE scale is associated with being unfocussed, untidy and with a low responsibility for schoolwork, which is stated as a clinical deviant score. On the SI scale, a high score (clinical deviant) is associated with a shy and introvert personality in social situations. An increased score on the RE scale is stated as a clinical deviant score and is associated with a postponement and avoidance trait with little or no responsibility regarding one’s own actions. People who obtain clinical deviant scores on this scale are described to behave selfishly, are distrustful, or reject others and feel indignant. On the DO scale, a high score represents a dominant personality trait and a lower score a dependent personality trait. Both scores are stated as a clinical deviant score.

### Statistical Analyses

First, Spearman’s rho correlation between age at implantation, speech perception, and language for the total CI group (*n* = 59) was computed. Next, for speech perception, subjects were categorized in a ‘high speech perception subgroup’ (speech perception score ≥ 85%) (*n* = 38) or ‘low speech perception subgroup’ (speech perception score < 85%) (*n* = 18). For language comprehension, subjects were categorized in ‘adequate language comprehension subgroup’ with a *z*-score of ≥ -1.00 (*n* = 17) or ‘poor language comprehension subgroup,’ with a *z*-score of < -1.00 (*n* = 39). PPVT and reading outcome scores were distributed evenly over the subgroups.

Statistical analyses were performed using IMB SPSS Statistics 22. For each test, the level of statistical significance was set at 5%. The percentage average, positive, or clinical deviant scores of the total group and subgroups were computed. The non-parametric Chi-Square test for Goodness of Fit was used to compare the distributions of scores of the adolescents with CI with the distribution of the reference group of the NPV-J. For the reference group, the percentage that performs below average (clinical deviant) is 15%, the percentage that performs within the average range is 70%, and the percentage that performs above average (positive deviant) is 15%. The effect size was measured using Cohen’s *w.*

First, the distributions of average and deviant scores on each personality trait of the total CI group were compared to those of the reference group. Next, the distributions for the personality test scores for the CI subgroups (speech perception high/low; language comprehension adequate/poor) were compared to the reference group of the NPV-J.

## Results

### Speech Perception and Language Comprehension

**FIGURE 1:**
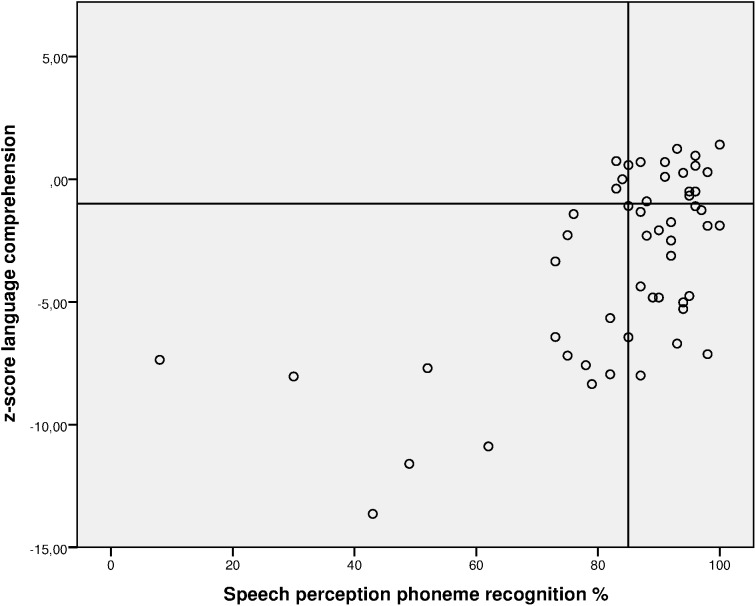
Scatterplot of percentage speech perception scores and language comprehension for the total CI group. Adequate performance on phoneme recognition ≥ 85%, on language comprehension *z* ≥ –1.00.

Significant correlations were found between age at implantation and speech perception; *r*_s_ = -0.540, *p* = 0.000, two-tailed, *n* = 56, between age at implantation and language comprehension; *r*_s_ = -0.463, *p* = 0.000, two-tailed, *n* = 56, and between speech perception and language comprehension; *r*_s_ = 0.534, *p* = 0.000, two-tailed, *n* = 53 for the total CI group. **Figure 1** shows the scatter plot of speech perception and language comprehension. Note only a small number of subjects had adequate language comprehension scores in the absence of a high speech perception score.

### Personality Scales

The adolescents with CI showed significant different distributions of average or deviant scores compared to the hearing reference group, on two personality scales: PE and RE. No significant different distributions of scores were found between the reference group and the children with CI on the other personality scales: IN, SI, and DO.

In this Section “Personality Scales,” we present the results for the total CI group, followed by the results according to the speech perception and language comprehension subgroups. First, we describe positive deviations from the reference group on the PE scale (i.e., in favor of the HI adolescents with CI). Second, we report data with negative (clinical) deviations from the reference group on the RE scale.

#### Positive Deviations on the Personality Scale Perseverance

The total CI group showed a significant higher proportion of positive deviant scores compared to the reference group on the personality scale PE, χ^2^ (2, *n* = 59) = 14.89, *p* < 0.05. The effect size was large (*w =* 0.50). This means that significant more adolescents with CI (31%) obtained an above average positive score on this scale compared to the reference group.

According to the speech perception subgroups, the high speech perception subgroup had a significantly higher proportion of positive deviant scores (26%) compared to the reference group, χ^2^ (2, *n* = 38) = 7.13, *p* < 0.05, with a medium effect (*w* = 0.43). Also, a significantly higher proportion of positive deviant scores was found for the low speech perception subgroup (39%) compared to the reference group on the personality trait PE, χ^2^ (2, *n* = 18) = 8.46, *p* < 0.05, with a large effect (*w* = 0.69).

There was no significant difference between the adolescents with CI with adequate language comprehension scores and the reference group. The poor language comprehension subgroup showed significantly higher proportion of positive deviant scores compared to the reference group on the personality scale PE, χ^2^ (2, *n* = 39) = 15.77, *p* < 0.05. The effect size was large (*w* = 0.64). This means that significantly more adolescents with CI with poor language comprehension scores (36%) obtained an above average positive score on this scale compared to the reference group.

**FIGURE 2:**
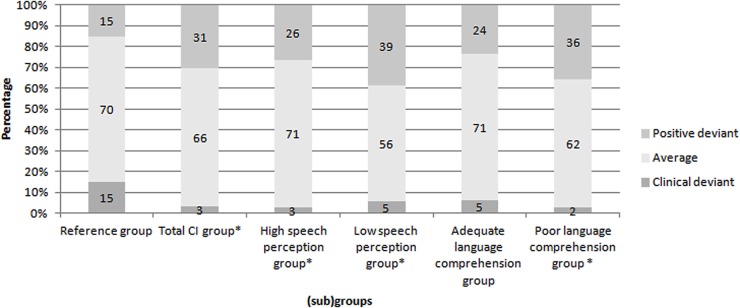
Distributions of the Perseverance scale scores of the reference group, the total CI group and the subgroups. ^∗^*p* < 0.05. Percentages of the distribution of scores for the reference group are depicted, as well as the percentages of the CI (sub)groups.

**Figure 2** displays the distributions of the PE scale scores for the reference group, the total CI group, and the CI subgroups. Percentages of the distribution of scores for the reference group are depicted, as well as the percentages of the CI (sub)groups.

#### Clinical Deviations on the Personality Scale Recalcitrance

The total CI group showed a significantly higher proportion of clinical deviant scores (29%) compared to the reference group on the personality scale RE χ^2^ (2, *n* = 59) = 12.85, *p* < 0.05. The effect size was medium to large (*w =* 0.47). Compared to the reference group, the proportion of subjects classified with clinical deviant RE scores did not significantly differ for the subgroup with high speech perception scores, whereas there was a difference for the subgroup with low speech perception scores χ^2^ (2, *n* = 18) = 12.50, *p* < 0.05. The effect size was large (*w* = 0.83). The low speech perception subgroup showed a significantly higher proportion of clinical deviant scores (44%) compared to the reference group.

There was no significant difference in RE scores between de reference group and the adolescents with CI and adequate language comprehension scores. There was, however, a significant difference in RE scores between the reference group and adolescents with CI with poor language comprehension scores, χ^2^ (2, *n* = 39) = 12.95, *p* < 0.05. The effect size was large (*w =* 0.58). The poor language comprehension subgroup showed a significantly higher proportion of clinical deviant scores (33%) compared to the reference group on this scale.

**FIGURE 3:**
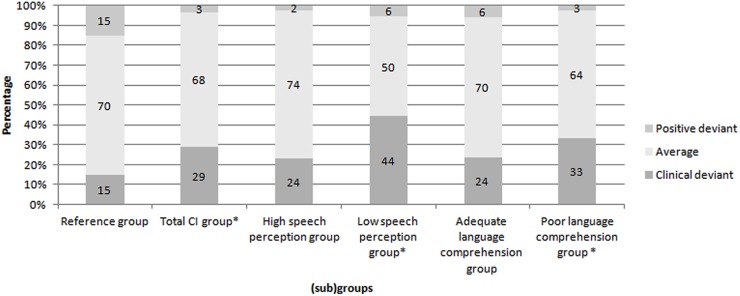
Distributions of the Recalcitrance scale scores of the reference group, the total CI group and the subgroups. ^∗^*p* < 0.05. Percentages of the distribution of scores for the reference group are depicted, as well as the percentages of the CI (sub)groups.

**Figure 3** displays the distributions of the RE scale scores for the reference group, the total CI group, and the CI subgroups. Percentages of the distribution of scores for the reference group are depicted, as well as the percentages of the CI (sub)groups.

## Discussion

The aim of the present study was to compare the personality traits of adolescents with CI to a reference group (normal-hearing peers). In addition, this study aimed to investigate differences between the reference group and CI subjects on personality traits according to level of speech perception skills (high/low) and level of language comprehension (adequate/poor). This study was motivated by previous research that has shown that HI adolescents with hearing aids are at risk of developing problems with social emotional and psychosocial development. HI adolescents therefore were vulnerable for developing personality disorders. For instance, Hindley et al. (1994) report in a prevalence study of psychiatric disorders in deaf children and adolescents a percentage of 50.3%. HI limits a child’s access to understanding and developing complex language skills, which could mediate personality development. With CI (implanted in childhood), prelingually profound HI adolescents have auditory access to speech and, in most cases, to levels of spoken language. This subsequently gives them the opportunity to develop higher level language and improved social communication skills which facilitates social learning, a prerequisite for developing a balanced personality.

The results of the present study show that adolescents with CI showed normal or favorable distributions on four of the five investigated personality traits (IN, SI, DO, and PE). Only for the RE trait, the total CI group, the subgroup with low speech perception scores and the subgroup with poor language comprehension scores showed a larger proportion of scores below average as compared to the reference group. Which means that more children with CI especially the CI children with low speech perception scores and poor language comprehension scores show a postponement and avoidance personality trait with little or no responsibility regarding one’s own actions. As hypothesized, adolescents with HI implanted with a CI who demonstrate high speech perception scores and adequate language comprehension scores showed similar distributions to normal hearing peers on all personality traits.

Indeed, good speech perception appeared to be a factor in the development of personality among adolescents. This finding is in line with the study by Nasralla et al. (2009). The subjects who obtained low speech perception results in the study by Nasralla et al. (2009), reported difficulties in the area of interpersonal contacts, reacting according to the affective and auditory situations, and did not reach their potential compared to the group with the high speech perception scores.

It is clear that hearing loss itself is not the only risk factor for experiencing social and emotional problems and problems in personality development. It appears that lack of language contributes to these problems (Stevenson et al., 2010; Gentilli and Holwell, 2011). In our study, adolescents with CI attain normal distributions on all personality scales if language comprehension skills were at an average or higher level. Stevenson et al. (2010) endorsed the idea that language is a significant factor in the psychosocial development of adolescents. The authors stated that hearing loss is related to an increased rate of behavior problems because hearing loss is a risk factor for low language competence.

Ketelaar et al. (2015) specifically examined the factor language and reported that emotional language is related to social functioning among children with CI and that language skill levels were related to the frequency of behavioral problems. In hearing children with language disorders, difficulties with social emotional functioning and behavioral adjustment exist not due to the HI. Language is known to support emotional self-regulation and social-cognitive competence. Several studies indicate that young people with specific language impairment are more likely to exhibit abnormal levels of emotional and behavioral difficulties than hearing peers (Toppelberg and Shapiro, 2000; Im-Bolter and Cohen, 2007; Durkin and Conti-Ramsden, 2010; Yew and O’Kearney, 2013). In our study, 66% of the subjects with a CI had high speech perception scores, but nonetheless, 40% had low language comprehension despite good hearing levels. These are children in which language or learning disorders may be present in addition to the hearing loss (Norbury et al., 2001).

In our study, adolescents with low speech perception scores, poor language comprehension scores, or both, frequently had clinical deviant scores on the RE scale. As stated in the Section “Introduction,” the unpredictability of actions based on lack of auditory information or misinterpretations in communication might result in suspicion and lack of trust. Mainly for children with UCI, auditory and language skills remained limited and are expected to have caused more dysfunctional RE traits than in the norm group.

Research of Geers et al. (2013), shows that well-developed social skills are more associated with the ability to discriminate the nuances of talker identity and emotion than with the ability to recognize words and sentences through listening. They found that both abilities were better in BICI children than in UCI children. This could be a secondary benefit of binaural hearing with BICI (Sparreboom et al., 2014). However, due to the small sample size of BICI children in our study, we were not able to perform analyses between these groups.

A positive finding in our study is that the adolescents with CI did not differ from the reference group in terms of the distributions of average or deviancy scores on the scales IN, SI, and DO. In support of this interpretation, other studies reported that children with CI could obtain average social skills and self-esteem in comparison to normal hearing peers. Children with CI showed comparable auditory levels as children with hearing aids, in addition to lower levels of behavioral problems than children with a hearing aid. The CI group showed equal empathy and social competence as normal hearing peers (Ketelaar et al., 2013; Theunissen et al., 2014). No differences in self-esteem and number of friends between children with CI and hearing peers were reported (Percy-Smith et al., 2008). Bat-Chava et al. (2005) found that children with CI demonstrated a rapid development in socialization with hearing peers after implantation.

Similarly, the level of auditory and language skills of or our study group does not hinder them in social interactions, subjectively. Hence, the adolescents’ answers to the questionnaire imply that they experience that they are able to comprehend social situations and that they feel secure. This is reflected in a normal personality trait development of (Social) IN.

This social safety also enables them to comply with situations rather than to control them, which is reflected in a normal DO personality trait.

Remarkable results were found on the scale PE. The total group of adolescents with CI as well as both speech perception subgroups and the subgroup with poor language comprehension scores obtained positive deviant scores more frequently compared to the reference group. The subgroup with adequate language comprehension scores did not show this favorable difference on the PE trait. Adolescents with CI with poor language comprehension scores obtained positive deviant scores more frequently compared to the reference group. It might be the case that these children are rewarded for effort rather than for good performance. Percy-Smith et al. (2008) reported that boys with CI were better in managing schoolwork and Wheeler et al. (2007) found that children with CI seek support to achieve mainstream goals.

## Conclusion

In conclusion, the findings of this study showed that personality traits that reflect social relations, self-conscience, and school- and task orientation in adolescents with CI are similar to those in normal-hearing peers. This finding holds despite variations in speech perception ability and language comprehension levels for the CI group. On the RE trait, however, adolescents with low speech perception and/or poor language comprehension scores more frequently obtained clinical deviant scores. This is an important factor to consider for both schools and services guiding these young adults. The adolescents in our study were implanted at a relatively late age compared to modern standards. Late age at implantation is associated with poorer speech perception and poorer language comprehension. Early (bilateral) implantation is expected to have a further positive effect on the development of personality traits of profoundly hearing impaired children predominately as a result of improved spoken language.

## Ethics Statement

This study was carried out in accordance with the Dutch ethical standards for University Medical Centres. Written informed consent from all subjects was obtained in accordance with the Declaration of Helsinki.

## Author Contributions

ML and AV formulated the research question. ML and AV coordinated the clinical data collection. MB was involved in data collection. All authors provided contributions in the analysis and interpretation of the data. MB drafted the manuscript, and AV, HM, and ML provided critical revisions. All authors approved the final version of the manuscript for submission.

## Conflict of Interest Statement

The authors declare that the research was conducted in the absence of any commercial or financial relationships that could be construed as a potential conflict of interest. The reviewer DT and the handling editor declared their shared affiliation.
